# Anterior cervical discectomy and fusion to treat cervical instability with vertigo and dizziness: A single center, retrospective, observational study

**DOI:** 10.3389/fsurg.2022.1047504

**Published:** 2023-01-06

**Authors:** Huo-Liang Zheng, Bo Li, Shao-Kuan Song, Peng-Bo Chen, Lei-Sheng Jiang, Sheng-Dan Jiang

**Affiliations:** ^1^ Department of Clinic of Spine Center, Xinhua Hospital, Shanghai Jiaotong University School of Medicine, Shanghai, China; ^2^ Department of Clinical Medicine, Shanghai Jiaotong University School of Medicine, Shanghai, China

**Keywords:** cervical spine, anterior cervical discectomy and fusion, vertigo, dizziness, instability

## Abstract

**Purpose:**

The current study attempts to investigate the role of anterior cervical discectomy and fusion (ACDF) in alleviating symptoms in patients with cervical vertigo associated with cervical instability.

**Methods:**

The patients of cervical instability with vertigo and dizziness who underwent ACDF between January 2011 and December 2019 were followed-up for more than two years. Demographic data (age, sex, follow up period and levels of instable cervical segments) were assessed; Symptoms of vertigo and dizziness before and after surgery were assessed by the 15-item version of the Vertigo Symptom Scale (VSS) and the 25-item Dizziness Handicap Inventory (DHI). The severity and frequency of other symptoms like neck and occipital pain, gastrointestinal discomfort, nausea, vomiting, tinnitus, palpitations, headache, diplopia and blurring of vision before and after surgery were also assessed.

**Results:**

A total of 92 patients underwent ACDF for cervical instability with vertigo and dizziness between January 2011 and December 2019, of which 79 patients were included in the final analysis. The number of instable levels had no correlation with VSS and DHI scores before surgery (*p* > 0.05), while patients with C3/4 instability suffering a severer vertigo than other levels. Both DHI and VSS scores were significantly reduced after ACDF and this was sustained within two years after surgery (*p* < 0.001). Although there was no statistical difference in the ratio of patients with vertigo relief, patients with one-level cervical instability demonstrated a more rapid recovery than patients with multi-level cervical instability (*p* = 0.048). Also, there was improvement in other symptoms such as neck and occipital pain, gastrointestinal discomfort, nausea, vomiting, tinnitus, palpitations, headache and blurring of vision after surgery.

**Conclusions:**

Vertigo caused by C3/4 instability was severer than other levels such as C4/5 and C5/6. During 2 years' follow-up the significant relief of vertigo and dizziness was observed after anterior cervical surgery. Other accompanying symptoms except hypomnesia were also extenuated in follow-up period.

## Introduction

Vertigo is “an illusion of movement”, and it may be rotational, oscillating or tilting in nature. Dizziness can be described as light-headedness, imbalance, giddiness, or unsteadiness, and it is perhaps closest to the definition of vertigo. There are a number of different causes of vertigo including central nervous system and central or peripheral vestibular dysfunction etc. Some patients are suspected that the cause of their problem is a disorder of the cervical spine, known as cervical vertigo (1). In 1955, Ryan and Cope used the term “cervical vertigo” to refer to a combination of cervical spine problems and dizziness (2). It is defined as vertigo induced by changes of position of the neck or vertigo originating from the cervical region. A proportion of patients having cervical instability can complain about varying degrees of symptoms of vertigo and dizziness without myelopathy and/or radiculopathy, and always accompanied by tinnitus, blurred vision, headache, nausea, vomiting, palpitations, and gastrointestinal discomfort etc. The pathophysiology behind the association of these clinical symptoms with mechanical problem is not very clearly known.

Anterior cervical discectomy and fusion (ACDF) is a commonly used approach for cervical instability (3). However, the effect of ACDF on these symptoms is yet to be explored. In this retrospective study, we aimed to investigate whether ACDF is effective in improving vertigo, dizziness, and these accompanied symptoms by comparing their severity before and after surgery.

## Materials and methods

### Patients

**Figure 1:**
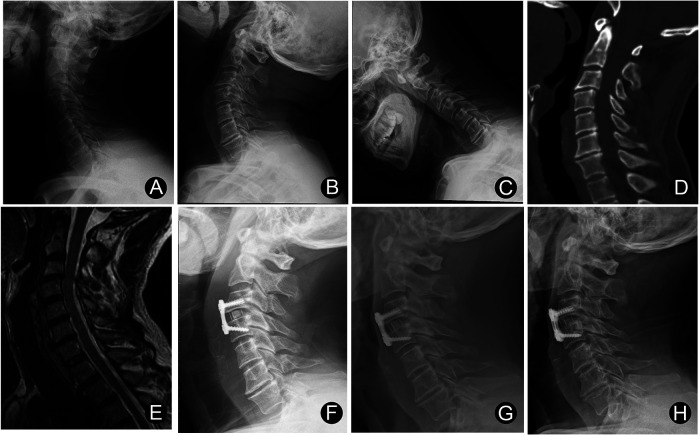
Male, 57 years old: C3/4 ACDF was experienced. (**A**–**C**) Cervical spine x-ray showing C3/4 instability. (**D**) Preoperative cervical CT sagittal image. (**E**) Magnetic resonance imaging (MRI) showing no significant compression of the spinal cord. (**F**) x-ray of cervical spine 3 months after surgery. (**G**) x-ray of cervical spine 12 months after surgery. (**H**) x-ray of cervical spine 24 months after surgery.

**Figure 2:**
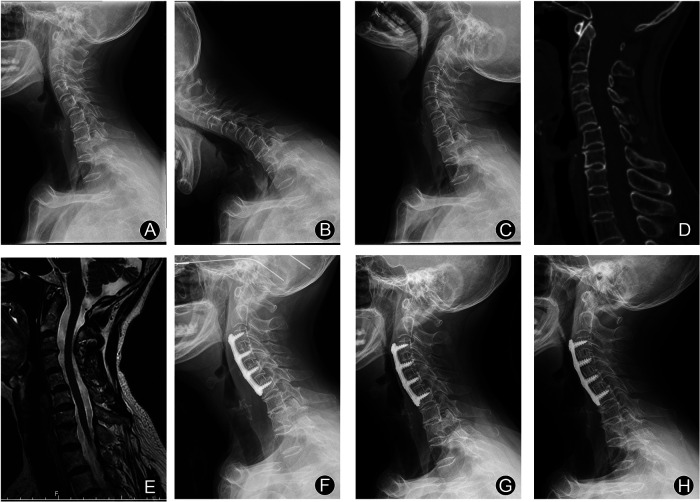
Female, 68 years old: three levels of ACDF was experienced. (**A**–**C**) Cervical spine x-ray showing C3/4, C4/5 and C5/6 instability. (**D**) Preoperative cervical CT sagittal image. (**E**) Magnetic resonance imaging (MRI) showing no significant compression of the spinal cord. (**F**) x-ray of cervical spine 3 months after surgery. (**G**) x-ray of cervical spine 12 months after surgery. (**H**) x-ray of cervical spine 24 months after surgery.

From January 2011 to December 2019, ninety-two cervical instability patients with vertigo and dizziness underwent ACDF with PEEK cages in our institution. Of these patients, 79 were available for follow-up evaluation for more than 2 years after surgery. All patients were followed up at least three times postoperatively, at three months, one year, and two years after surgery. The mean of last follow-up was 29.6 months (range: 24 to 96 months). There were 14 males and 65 females. The ages ranged from 49 to 82 years, with a mean of 67.4 years. For patients with only one level of cervical instability, we performed single-level ACDF (Figure 1). For patients with two or more cervical instability, we performed ACDF on the corresponding segments (Figure 2). At the follow-up, patients underwent postoperative cervical spine x-ray (anteroposterior and lateral projections) and assessment of clinical symptoms such as vertigo, dizziness, neck and occipital pain and so on. This study included patients who had 2 years' follow-up visit after surgery with the complete results of clinical and radiological assessments.

### Inclusion and exclusion criteria

The inclusion criteria were as follows: (1) all patients presented vertigo and dizziness without myelopathy or radiculopathy. (2) Flexion-extension x-rays were used to assess stability of the cervical spine, and sagittal translation (>3.5 mm), or segmental angulation (>11°) was typically used to diagnose cervical instability (4). (3) Obvious cervical spinal cord compression was not demonstrated on magnetic resonance imaging (MRI). (4) Diseases relating to neurology, otolaryngology, ophthalmology, and cardiovascular diseases such as Meniere disease, cataract, lacunar infarcts, etc., were excluded. (5) The conservative treatment is ineffectual, and all the patients underwent ACDF. The exclusion criteria were as follows: (1) Alternative etiology of vertigo and dizziness revealed on consultation with neurology, otolaryngology, cardiology or ophthalmology. (2) A history of cervical spine trauma or surgery.

## Methods

All the patients underwent a clinical evaluation. A cervical spine examination was mainly performed to assess cervical mobility by standard flexion-extension x-ray imaging. A neurological assessment was completed by the brain MRI examination and evaluating of the strength of the four limbs, surface and deep sensitivity and coordination. In addition, a comprehensive ENT examination including an electronystagmogram was used in order to rule out potentially balance-altering vestibular damage. In the absence of abnormal clinical examination results, we considered that a patient's vertigo and dizziness was non-vestibular.

Perceived frequency and severity of vertigo and dizziness was assessed by the 15-item version of the Vertigo Symptom Scale (VSS) (5). The scale has 5 response categories (0–4). Total scale scores range between 0 and 60 points, severe dizziness: ≥12 points. clinically significant change: ≥3 points.

Perceived disability was assessed by the 25-item Dizziness Handicap Inventory (DHI) which has 3 response categories (0; 2; 4). Total scores range from 0 to 100 points (23), interpreted as mild 0–30; moderate 31–60; severe 61–100 (6).

To evaluate the outcome of surgery, the closest minimally clinical important difference (MCID) in terms of follow-up was used 11 for the VSS and 17 for DHI at the term of 2 years.

In addition to vertigo and dizziness, other symptoms such as neck and occipital pain, gastrointestinal discomfort, nausea, vomiting, tinnitus, palpitations, headache, hypomnesia, diplopia and blurring of vision before and after surgery were also recorded.

As there is no standardized method to assess the severity and frequency of neck and occipital pain, gastrointestinal discomfort, nausea, vomiting, tinnitus, palpitations, headache, hypomnesia and blurring of vision, we used a scale to objectively record the data. The outcomes were the intensity and frequency of these symptoms. The intensity was measured with a 100 mm visual analogue scale (VAS). Total scale scores range between 0 and 100 points, clinically significant change: ≥10 points.

### Statistical analysis

Quantitative information is presented as mean and standard deviation. The Shapiro–Wilk test was used to test the normality of continuous data. One-way repeated measures analysis of variance (ANOVA) was used to compare the indicators in the same group at different time points, while Friedman test was used for data that does not fit Gaussian distribution. Multiple comparison analysis between groups was analyzed using least significant difference (LSD). Binary and categorical indicators between groups were compared using the exact two-way Fisher criterion. The comparison of categorical variables before and after surgery was performed using the McNemar criterion. Binary logistic regression was used to investigate whether age, sex and surgery in different cervical levels influences the amelioration of vertigo and dizziness. The log-rank criterion was used to analyze the relief of vertigo after single-level and multi-levels cervical spine surgery in three weeks. Statistical significance was defined by *p* < 0.05. Data were analyzed using SPSS software version 21 for Windows 11.

## Results

### General results

A total of 79 patients underwent ACDF for cervical instability with cervical vertigo and dizziness were included in the final analysis. The main characteristics of patients are presented in Table 1. Most of the patients with cervical instability (65/79, 82.3%) were female. Among the patients, the majority had one-level cervical instability (60/79, 75.9%). The most common level of instability was C3–4 (*n* = 46, 46.0%) followed by C4–5 (*n* = 40, 40.0%) and C5–6 (*n* = 14, 14.0%). There was no significant association between the number of instability levels and symptoms of vertigo and dizziness as measured by VSS (*p* = 0.724) and DHI (*p* = 0.780) (Table 2). Vertigo and dizziness caused by the instability of C3/4 are significantly worse than those caused by C4/5 or C5/6, as evidenced by VSS and DHI scores (Table 3).

**Table 1:** Basic data of patients (x ±, *n* = 79).

Characteristics	No. of patients (*n* = 79)
Age at surgery	67.4 center8.2
Sex	
Male	14 (17.7%)
Female	65 (82.3%)
Levels of instability segments	
One-level	60 (75.9%)
Two-levels	17 (21.5%)
Three-levels	2 (2.5%)
Numbers of surgical segments	
C3/4	46 (46.0%)
C4/5	40 (40.0%)
C5/6	14 (14.0%)
follow-up period, months	29.6 ± 9.6

**Table 2:** Preoperative VSS and DHI scores in patients with different numbers of instability segments (x ±, *n* = 79).

Parameters	One-level	Two-levels	Three-levels	F	*p*
VSS	22.8 ± 4.0	22.2 ± 4.5	24.5 ± 0.7	0.324	0.724
DHI	37.8 ± 4.7	38.5 ± 3.4	39.5 ± 2.1	0.250	0.780

VSS, Vertigo Symptom Scale; DHI, Dizziness Handicap Inventory.

**Table 3:** Preoperative VSS and DHI scores in patients with different cervical segments (x ±, *n* = 79).

Parameters	C3/4 (*n* = 32)	C4/5 (*n* = 21)	C5/6 (*n* = 7)	C3-5 (*n* = 12)	C4-6 (*n* = 5)	C3-6 (*n* = 2)	F	*p*
VSS	24.4 ± 3.5	21.4 ± 4.0^a^	19.9 ± 3.2^b^	22.2 ± 3.7	22.4 ± 6.6	24.5 ± 0.7	2.63	0.031
DHI	39.7 ± 4.6	36.0 ± 4.3^a^	34.9 ± 2.8^b^	38.4 ± 3.7	38.6 ± 3.2	39.5 ± 2.1	2.91	0.019

Note: Comparison of preoperative VSS and DHI scores between C3/4 and C4/5.

^a^ *p* < 0.05; Comparison of preoperative VSS and DHI scores between C3/4 and C5/6.

^b^ *p* < 0.05.

### Vertigo and dizziness

**Figure 3:**
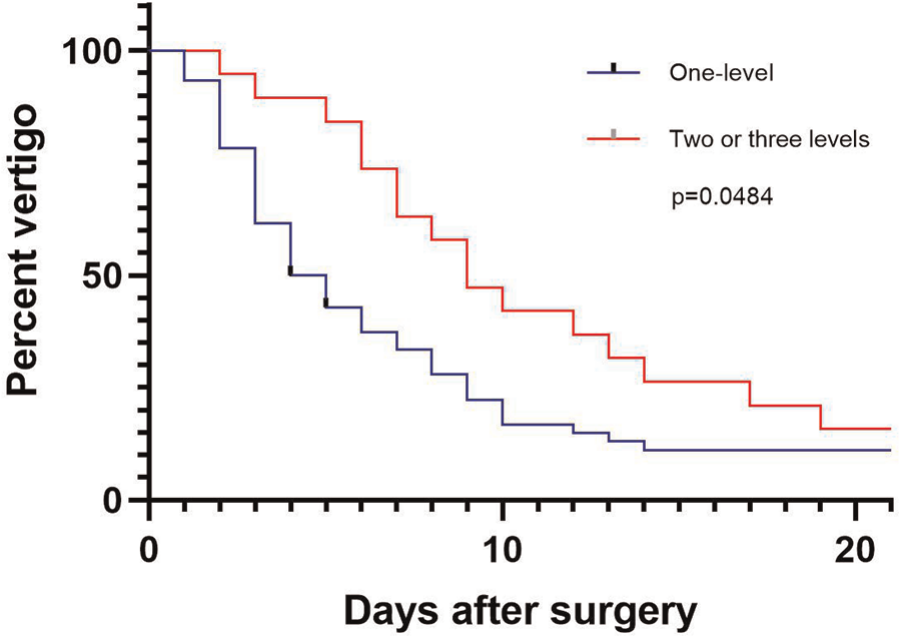
Percentage of patients with residual vertigo within three weeks after ACDF.

Vertigo and dizziness assessed by DHI and VSS were significantly relieved after ACDF and this was sustained at the final follow-up (Table 4). In fact, within 10 days after surgery, the relief of vertigo and dizziness was observed. Compared with single-level ACDF, two or three levels ACDF has slower symptom relief (Figure 3). About half of patients experienced significant improvement in vertigo within 4 days after single-level ACDF, while it extended to 9 days in multi-level ACDF. Although means of VSS and DHI scores decreased obviously at 12 and 24 months compared with those at 3 months, there was no statistically significant difference between scores at 12 months and 24 months after surgery.

**Table 4:** VSS and DHI scores before and after surgery (x ±, *n* = 79).

Parameters	before surgery	3 months after surgery	12 months after surgery	24 months after surgery	F	*p*
VSS	22.7 ± 4.0	6.8 ± 4.0^a^	5.8 ± 2.9^a^	5.4 ± 2.7^a^	140.7	<0.001
DHI	38.0 ± 4.4	13.8 ± 4.7^a^	13.0 ± 3.7^a^	12.9 ± 3.8^a^	640.3	<0.001

Note: Comparison of VSS and DHI scores between before surgery and after surgery.

^a^ *p* < 0.05.

**Figure 4:**
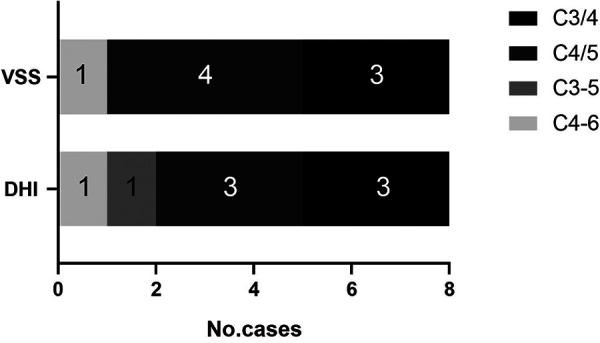
Distribution of levels of cervical spine failing to meet MCID assessed by VSS and DHI.

As an effective method of assessing the validity of scale, MCID is earning its place and recognition by patients and clinical doctors. Consequently, we evaluated the alleviating effects of ACDF on vertigo and dizziness using the MCID of VSS and DHI scales. As shown in Table 5, most patients (89.9%) achieved MCID after surgery and there is no significant difference in the ratio of achieving MCID between patients accepting one-level ACDF and multi-level ACDF. Figure 4 shows the number of patients who failed to achieve MCID at different cervical levels as measured by VSS and DHI scores.

**Table 5:** Patient-reported outcomes during follow-up terms (*n* = 79).

Parameter	Levels of instability segments		*n*	*p*
	One-level	> One-level		
VSS				
Achieved MCID	53 (88.3%)	18 (94.7%)	71 (89.9%)	0.672
Not achieved MCID	7 (11.7%)	1 (5.3%)	8 (10.1%)	
DHI				
Achieved MCID	54 (90.0%)	17 (89.5%)	71 (89.9%)	>0.99
Not achieved MCID	6 (10.0%)	2 (10.5%)	8 (10.1%)	

MCID, Minimally clinical important difference.

Furthermore, we investigated factors influencing the efficacy of ACDF on vertigo according to whether the MCID of VSS and DHI were both achieved. Although the preoperative scores of VSS and DHI varied with the levels of instable cervical segments, logistic regression showed that there was no significant correlation between postoperative amelioration of vertigo and the level of instable cervical segments such as involving C3/4 (OR = 0.386, *p* = 0.338), involving C4/5 (OR = 1.199, *p* = 0.849) and C5/6 (OR = 1.027, *p* = 0.980) (Table 6). Also, the correlation between postoperative amelioration of vertigo, age (OR = 1.046, *p* = 0.339) and sex (OR = 0.457, *p* = 0.371) was not observed.

**Table 6:** Odds ratio, 95% CI and *P* value association using multiple factors logistic regression models for vertigo meeting the MCID including cervical levels.

Parameter	Odds ratio	95% CI	*p*
C3/4 (involved)	0.386	(0.055, 2.706)	0.338
C4/5 (involved)	1.199	(0.185, 7.785)	0.849
C5/6 (involved)	1.027	(0.133, 7.930)	0.980
Age	1.046	(0.954, 1.148)	0.339
Sex	0.457	(0.082, 2.536)	0.371

### Distribution of accompanying symptoms

The distribution of symptoms has been illustrated in Table 7. Besides vertigo and dizziness, out of 79 patients, blurring of vision (59/79, 74.7%) was the most common accompanying symptoms followed by tinnitus (58/79, 73.4%) and headache (56/79, 70.9%) before surgery. All accompanying symptoms were obviously extenuated 3 months after ACDF except hypomnesia (Table 8). Compared to preoperative symptoms, treatment with ACDF surgery has a significant effect on reducing the ratio of patients with neck and occipital pain (*p* < 0. 001), gastrointestinal discomfort (*p* = 0. 007), nausea (*p* = 0.004), tinnitus (*p* < 0.001), vomiting (*p* = 0.003), palpitation (*p* < 0.001), headache (*p* < 0.001) and blurred vision (*p* < 0.001). Diplopia in two patients disappeared after surgery. Although most patients experienced symptom relief after surgery, there was no significant effect on the relief of hypomnesia (*p* = 0.302) in patients experiencing ACDF (Tables 9–11).

**Table 7:** Number and incidence of other symptoms accompanied with vertigo.

symptoms	No. cases	Percent (%)
vertigo and dizziness	79	100
neck and occipital pain	53	67.1
gastrointestinal discomfort	35	44.3
nausea	29	36.7
vomiting	17	21.5
tinnitus	58	73.4
palpitations	43	54.4
headache	56	70.9
hypomnesia	37	46.8
diplopia	2	2.5
blurring of vision	59	74.7

**Table 8:** Severity of symptoms accompanied with vertigo before and after surgery (x ±).

Parameter	Before surgery	3 months after surgery	12 months after surgery	24 months after surgery	F	*p*
Neck and occipital pain (*n* = 53)	38.0 ± 7.7	13.9 ± 6.7^a^	10.8 ± 5.5^b^	9.3 ± 4.6	303.1	<0.001
Gastrointestinal discomfort (*n* = 35)	38.9 ± 6.5	11.0 ± 4.7^a^	10.7 ± 4.6	10.3 ± 5.1	337.9	<0.001
Nausea (*n* = 29)	31.4 ± 6.1	11.2 ± 4.3^a^	9.9 ± 4.1	10.0 ± 4.3	162.1	<0.001
Vomiting (*n* = 17)	36.7 ± 5.6	6.6 ± 3.7^a^	5.7 ± 3.3	5.0 ± 2.6	306.9	<0.001
Tinnitus (*n* = 58)	29.4 ± 4.4	11.9 ± 4.6^a^	10.7 ± 4.4	9.6 ± 4.6	339.4	<0.001
Headache (*n* = 56)	42.4 ± 7.3	11.8 ± 5.6^a^	10.4 ± 5.7	10.0 ± 5.3	461.7	<0.001
Blurring of vision (*n* = 59)	46.8 ± 8.3	16.2 ± 7.3^a^	14.9 ± 7.5	14.0 ± 7.3	341.6	<0.001
Hypomnesia (*n* = 37)	12.3 ± 2.1	11.9 ± 3.8	10.9 ± 4.8	11.4 ± 4.7	1.69^FD^	0.6386
Palpitations (*n* = 43)	36.8 ± 6.3	13.4 ± 5.0^a^	12.1 ± 5.3	10.1 ± 5.2^c^	323.2	<0.001

Note: Comparison of symptoms accompanied with vertigo between before surgery and 3 months after surgery.

^a^ *p* < 0.05; Comparison of symptoms accompanied with vertigo between 3 months after surgery and 12 months after surgery.

^b^ *p* < 0.05; Comparison of symptoms accompanied with vertigo between 12 months after surgery and 24 months after surgery.

^c^ *p* < 0.05; FD, Friedman test.

**Table 9:** Number of patients with neck and occipital pain, gastrointestinal discomfort or blurring of vision before and after surgery. (x ±, *n* = 79).

Symptom	Neck and occipital pain (After surgery)		Gastrointestinal discomfort (After surgery)		Blurring of vision (After surgery)	
	Symptomatic	Symptomless	Symptomatic	Symptomless	Symptomatic	Symptomless
Symptomatic (Before surgery)	28	25	22	13	41	18
Symptomless (Before surgery)	1	25	2	42	2	18
*P*	<0.001		0.007		<0.001	

Note: Comparison of neck and occipital pain, gastrointestinal discomfort and blurring of vision before and after surgery.

**Table 10:** Number of patients with nausea, vomiting or tinnitus before and after surgery. (x ±, *n* = 79).

Symptom	Nausea (After surgery)		Vomiting (After surgery)		Tinnitus (After surgery)	
	Symptomatic	Symptomless	Symptomatic	Symptomless	Symptomatic	Symptomless
Symptomatic (Before surgery)	15	14	0	17	28	30
Symptomless (Before surgery)	3	47	3	59	1	20
*P*	0.004		0.003		<0.001	

Note: Comparison of nausea, vomiting or tinnitus before and after surgery.

**Table 11:** Number of patients with hypomnesia, headache or palpitations before and after surgery. (x ±, *n* = 79).

Symptom	Hypomnesia (After surgery)		Headache (After surgery)		Palpitations (After surgery)	
	Symptomatic	Symptomless	Symptomatic	Symptomless	Symptomatic	Symptomless
Symptomatic (Before surgery)	27	10	29	27	18	25
Symptomless (Before surgery)	5	37	3	20	2	34
*P*	0.302		<0.001		<0.001	

Note: Comparison of hypomnesia, headache or palpitations before and after surgery.

## Discussion

To date, the etiology and mechanisms of cervical vertigo are still unknown, conservative therapy has been the main treatment which has been unsatisfactory. Some studies (7–9) manifested that ACDF improved the sympathetic symptoms like vertigo, headache, nausea, vomiting and gastrointestinal discomfort in patients with cervical radiculopathy and/or myelopathy. This doesn't mean that all patients who have cervical spondylosis with concomitant vertigo and dizziness should be treated with anterior cervical surgery. Treatment of cervical vertigo is complicated in patients who have chronic neck pain and concomitant vertigo and dizziness but without cervical disc herniation or compression of nerve root and spinal cord.

In 1928, Pearce and Barré–Liéou (10) suggested that cervicogenic dizziness was due to an abnormal input from the cervical sympathetic nerves. They proposed that the posterior sympathetic plexus could be mechanically irritated by degenerative arthritis and induce reflex vertebrobasilar vasoconstriction and symptoms of vertigo and dizziness. It is well known that cervical spinal tissues are rich in sympathetic fibers. The cervical dura mater and the posterior longitudinal ligament have different sympathetic innervation patterns (11). In addition, the cervical sympathetic trunk consists of a main trunk and two to four ganglia which are located anterior to the transverse processes (12, 13). We speculated that abnormal motion of the cervical segment may stimulate the sympathetic nervous system other than the vertebral artery which induces symptoms such as vertigo, dizziness, tinnitus, nausea, vomiting, palpitations, headache, hypomnesia, and gastrointestinal discomfort.

Some authors have attributed cervical vertigo to the dynamic vertebrobasilar insufficiency (14, 15). In other words, at least in a subset of dizzy patients with degenerative cervical spine disorders, the cause of dizziness on turning the neck could be due to the reduced vertebral blood flow. The complementary tests used to diagnose vertebrobasilar insufficiency are still controversial. As a consequence of the fact that vertebral artery stenosis is transitory, the use of these tests in asymptomatic patients is usually negative. Vertebrobasilar insufficiency secondary to cervical instability may be a mechanism in patients with vertigo and dizziness in our study.

ACDF surgery contributing to segmental cervical vertebrae fixation and fusion seems to be an effective surgical treatment modality for alleviating vertigo, dizziness and other sympathetic symptoms caused by cervical instability. We included patients who have cervical instability with vertigo and dizziness, and the main symptoms are vertigo and dizziness but not neck pain in study. And our study revealed that symptoms of vertigo and dizziness relieved after anterior cervical surgery and the surgical results were encouraging.

MCID indicates minimum clinically important differences and is an important metric in evaluating resolution of symptoms (16, 17). However, there is no consensus on the MCID value for VSS and DHI. Emasithi A has reported 17 as the MCID of DHI-TH (Thai version of the Dizziness Handicap Inventory) (18). The MCID of VSS and DHI used in this study were obtained numerically by using anchor-based method. To better assess the effectiveness of ACDF surgery on vertigo and dizziness resolution, we divided patients into two groups depending on whether the MCID of VSS and DHI was achieved. About 90% patients get a satisfactory improvement in vertigo and dizziness while the number of surgical levels didn’t influence the symptom relief.

Also, there was improvement in severity and frequency of other symptoms such as neck and occipital pain, gastrointestinal discomfort, nausea, vomiting, tinnitus, palpitations, headache, diplopia and blurring of vision after surgery. Though the specific mechanism of ACDF in improving these symptoms are not clearcut, the anterior cervical surgery might be useful to reduce abnormal motion of the cervical segment which lead to the aberrant stimulation of sympathetic nerves.

Although our preliminary results are encouraging, long-term follow-up of the surgically treated cases are still needed. Moreover, randomized controlled studies are warranted to further investigate the surgical outcome of cervical vertigo.

In summary, the diagnosis and treatment of cervical vertigo still remain controversial. Patients with cervical instability may have symptoms of vertigo and dizziness, and successful clinical results in terms of symptom improvement can be obtained in such patients with anterior cervical surgery. Relief of vertigo and dizziness following anterior surgery can be attributed to stabilization of the cervical segment, the elimination of irritation of sympathetic plexus and vertebrobasilar insufficiency. With other causes of the symptoms dismissed, anterior cervical surgery becomes an option when conservative treatment fails.

## Limitations

Our present study has limitation. Few patients underwent three-level cervical surgery, these patients were included in the two-level group in partial analysis.

## Conclusion

The most severe symptoms of vertigo are caused by C3/4 instability and the number of levels of instability segments are not significantly influenced. The present study indicated that ACDF can relieve vertigo and dizziness caused by cervical instability and most of the accompanying symptoms could also be greatly extenuated.

## Data Availability

The raw data supporting the conclusions of this article will be made available by the authors, without undue reservation.
